# Authenticity in Olive Oils from an Empeltre Clonal Selection in Aragon (Spain): How Environmental, Agronomic, and Genetic Factors Affect Sterol Composition

**DOI:** 10.3390/foods11172587

**Published:** 2022-08-26

**Authors:** Raquel Rey-Giménez, Ana Cristina Sánchez-Gimeno

**Affiliations:** 1 Laboratorio Agroambiental, Gobierno de Aragón, Avda. Montañana 1005, 50071 Zaragoza, Spain; 2 Tecnología de los Alimentos, Facultad de Veterinaria, Universidad de Zaragoza, Instituto Agroalimentario de Aragón-IA2, Miguel Servet 177, 50013 Zaragoza, Spain

**Keywords:** olive oil, Empeltre clone, crop year, harvest date, sterol composition, authenticity

## Abstract

Sterol composition is used as a “fingerprint” to demonstrate the authenticity of olive oils. Our study’s objective was to exhaustively characterize the sterol composition of Empeltre olive oils from clonal selection during the ripening period in 2017, 2018, and 2019. We likewise assessed the influence of crop year, fruit ripening, and clonal selection on the oils’ regulatory compliance in terms of sterol composition. Empeltre olive oils were shown to have medium-range *β*-sitosterol and Δ5-avenasterol content, along with elevated amounts of campesterol and Δ7-stigmastenol. A total of 26% and 12% of the samples were non-compliant in terms of apparent *β*-sitosterol and Δ7-stigmastenol, respectively. Crop year was the most influential factor in the case of most sterols. Clone type was the least influential factor, except in the case of campesterol. Olive maturity was only significant for Δ7-sterols. We likewise applied a discriminant analysis, with “crop year” as the grouping variable: 94.9% of the oils were thereby classified correctly.

## 1. Introduction

Extra virgin olive oil (EVOO) is one of the edible vegetable oils most widely consumed in Mediterranean countries due to its health benefits [1,2] and its renowned organoleptic characteristics. These properties are due to the chemical composition of olive oil, of which the glyceric fraction is the majority (98–98.5%). The rest, or minority fraction, is constituted, among other components, of sterols, polyphenols, tocopherols, and pigments. These compounds provide the unique nature of olive oil.

Pre-and post-harvest factors [3,4] affect olive oil quality and chemical composition. Studies have been described concerning the variation in the composition of sterols [5], fatty acids [6], polyphenols [7], alcohols [8], or waxes [9].

Countries that are not traditional olive oil consumers, such as the US or Brazil, have considerably increased their level of consumption over the last ten years [10]. Consequently, EVOO is the oil that achieves the highest price on the markets and is thus highly susceptible to certain fraudulent activities that endanger its authenticity. Deliberate mislabeling or adulteration through illegal blending with other oils or fats of lower economic value are the most common practices [11]. To safeguard olive oils, a series of regulations have been developed that regulate the physicochemical and organoleptic characteristics of olive oils according to their commercial category, and these regulations must be respected according to the country in which the oils are marketed. Among the most important regulations, Regulation (ECC) No 2568/91 is mandatory in the European Union [12] under sanction, but the international standards of the International Olive Oil Council (IOC) [13] and Codex [14] are not. The cultivation of olive trees in countries with climates quite different from the Mediterranean area has led to the appearance of commercial regulations in such nations as well [15,16]. Those standards generally take natural variations in the ranges of certain chemical compounds, such as sterols, into account.

The total concentration and composition of sterols in vegetable oils depend on the type of fruit or oilseed; they also differ from animal fats [17]. Sterols (4-desmethylsterols) are the main chemical compounds naturally present in the unsaponifiable fraction of olive oil. The sterol profile is used as a “fingerprint” to verify the authenticity of olive oil and is considered a purity parameter, according to commercial standards [12,13,14,15]. Non-compliance with a single limit value specified in the standards presumably indicates illegal blending with oils other than olive oil. Testing for non-compliance is a mode of protecting olive oil authenticity. However, numerous cases have been reported where genuine single-varietal olive oils naturally exceeded these limits; as a consequence, their economic value has decreased. In Spain, for example, certain olive oils from cultivars such as Cornicabra and Empeltre exceed the limits [12] for campesterol [18,19,20] and Δ7-stigmastenol [21,22,23,24] (4.0% and 0.5%, respectively). Similar non-compliances have likewise been described in single-varietal oils from non-Mediterranean countries, such as Argentina [16], Australia [25,26], the United States [27], and Iran [28].

Natural variations in sterol composition influenced by genetics [29,30,31,32,33], environment (soil, location, climate, water) [19,25,27,28,30,34,35,36], fruit ripening stage [18,25,31,32,37,38], or technological factors [19,26] have been described. Such variations could at least partially explain the deviations of certain genuine olive oils from established regulatory limits.

Empeltre [21,39] is a traditional olive cultivar located in NE Spain, with an area of 70,000 ha [39]. This cultivar is widely grown in the region of Aragon and protected under a total of six Protected Designations of Origin (PDO), two of which are in Aragon. In 1998–2002, a clonal pre-selection [23,24] was carried out with the purpose of genetically improving this cultivar, taking into account, among other parameters, the sterol composition of the oils obtained from it, especially its Δ7-stigmastenol content. The selected clones were subsequently planted in two comparative trials, one of which was conducted in Aragon [40].

The aim of the present study is to exhaustively characterize the sterol composition of Empeltre olive oil from Aragon clonal selection to evaluate the influence of environmental, fruit ripening, and clonal selection factors, and to ascertain the regulatory compliance of total and individual sterol amounts.

## 2. Materials and Methods

### 2.1. Plant Material and Fruit Samples

Empeltre [23,41] is a variety with low rooting capacity. For this reason, it is normally propagated by grafting. This cultivar has a high and constant production every year. The fruit is black when ripe, medium-sized, elongated, and slightly asymmetrical. The fruits are early ripening.

Eight Empeltre clones of olive trees, grown in an experimental orchard of Centro Transferencia Agroalimentaria in Alcañiz in the province of Teruel (NE Spain; altitude 295 m; longitude 41°03′ N; latitude 0°08′ W), were selected based on region of origin (Aragon). Our study was carried out over three consecutive seasons: 2017, 2018, and 2019. All olive trees were cultivated under identical agronomic and pedoclimatic conditions. The clonal selection was planted in 2004 at a 6 × 5 m spacing in clay loam soil with a drip irrigation system according to evapotranspiration. Standard cultivation practices were followed, so olive trees were well supervised and showed no nutrient deficiency or pest damage.

**Figure 1:**
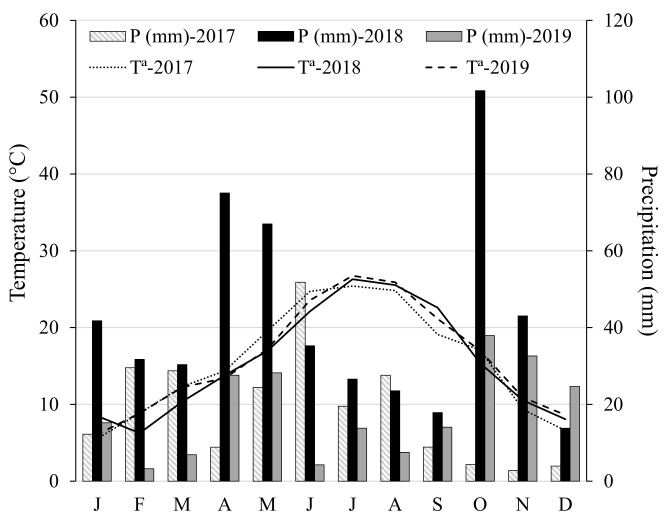
Mean values of temperature and rainfall registered in Alcañiz (Teruel) during three consecutive crop seasons (2017, 2018, and 2019). J: January; F: February; M: March; A: April; M: May; J: June; J: July; A: August; S: September; O: October; N: November; D: December.

The Alcañiz region has a cold, semi-arid climate (“BSk” type) according to the Köppen–Geiger climate classification [42], featuring irregular, scarce precipitation combined with wide absolute thermal amplitude owing to extreme temperatures in winter and summer. Figure 1 shows the weather data registered for the three years of study.

The clones we studied (3 trees per clone), numbered 1 to 8, came from a clonal pre-selection [23]. The clone identified in this study as “Std” corresponds to a group of clones comprising Nos. 1, 5, and 8, as a previous study observed no differences among them [40].

The olive fruits were hand-harvested at fortnightly intervals from October to December and were processed in the laboratory on the same day. The ripening index (RI) was determined according to the method described by Hermoso et al. [43] based on the color changes observed in the olives’ skin and pulp.

### 2.2. Olive Oil Extraction

Olive samples (4 kg) were processed, and their oil extracted using the two-phase Abencor^®^ laboratory oil mill (MC2, Ingeniería y Sistemas, S.L., Sevilla, Spain) [44]. The fruits were milled by a 3-mm-sieve stainless hammer mill, and the olive paste was malaxed for 30 min at 30 °C. The olive oils were then separated by centrifugation at 3500 rpm for one minute and further decanting. Finally, oils were filtered and the samples were stored in 125 mL amber glass bottles under a nitrogen atmosphere at −20 °C until analysis.

### 2.3. Determination of Physicochemical Quality Parameters

Free acidity, peroxide value, and ultraviolet (UV) absorption characteristics (K_232_, K_270_, ΔK) were determined according to methods described in Annexes II, III, and IX, respectively, of the consolidated European Regulation EEC No 2568/91 [11].

### 2.4. Determination of Sterol Composition

Sterol composition was determined according to the method described in Annex V of the consolidated European Regulation EEC No 2568/91 [11]. Sterol derivatives (trimethylsilyl ethers) were analyzed by an Agilent gas chromatograph (6890, Agilent, Santa Clara, CA, USA) equipped with a split/splitless injector (injection volume: 1 µL; split ratio: 1:50; 285 °C) and a flame-ionization detector (FID) (300 °C). Individual sterols were separated by a CP-Sil 8 CB capillary column (25 m length × 0.25 mm inner diameter × 0.25 µm film thickness) (Supelco, Bellefonte, PA, USA), and helium was used as a carrier gas (flow: 1 mL/min). The oven temperature was isothermal at 265 °C.

Individual peaks were identified by comparing the retention times of sterols with those of the standard samples. *α*-cholestanol was used as the internal standard for the quantification of individual sterols expressed as a relative percentage. The total sum of sterols was expressed as mg/kg. Apparent *β*-sitosterol was calculated as the sum of *β*-sitosterol, Δ5-avenasterol, clerosterol, sitostanol, and Δ5,24-stigmastadienol.

### 2.5. Statistical Analysis

We characterized the Empeltre clonal selection oils by descriptive analysis of all results obtained during the three years of study (78 samples). The effects of crop year, clone, and fruit maturity on individual and total sterol content were evaluated using univariate factorial analysis of variance (three-way ANOVA; *p* < 0.05). Clones 4 and 7 were excluded from three-way ANOVA analysis due to insufficient data. Results were grouped into three different ripening stages (green, spotted, and ripe) to evaluate the ripening effect. One-way ANOVA and a post hoc Duncan’s test (*p* < 0.05) were used to determine the influence of different harvest dates on sterol composition for each crop year. A study of relationships among individual sterols was carried out using Pearson’s correlation.

Finally, we conducted a multivariate analysis to study the discriminatory capacity regarding the sterol composition of Empeltre olive oils in the clonal selection of the study. For this purpose, we performed a canonical discriminant analysis (DA), which uses canonical correlation and principal component analysis techniques. To choose the most discriminating independent variables, we applied the step-wise method using Wilks’ lambda and its chi-square approximation as the exclusion method, along with the Snedecor’s F statistic as the selection criterion.

Statistical analyses were performed using IBM SPSS Statistics 24.0 software (IBM Corp., Armonk, NY, USA). Graphs were constructed with Excel 2016 (Microsoft Corp., Redmond, Washington, USA).

## 3. Results and Discussion

### 3.1. Sterol Composition and Regulatory Compliance of Empeltre Olive Oils

Table 1 shows the average results of the physicochemical quality parameters and the relative sterol composition, as well the total sterol content of the featured Empeltre olive oils. The 78 olive oil samples analyzed in the course of the 2017, 2018, and 2019 crop years were classified as extra virgin olive oils (EVOO) based on their physicochemical quality (acidity ≤ 0.8% oleic acid; peroxide value ≤ 20 meq O_2_/kg; K_270_ ≤ 0.22; K_232_ ≤ 2.50) according to the different regulations [12,13,14]. The low acidity levels we observed indicate that these olive oils came from healthy fruit.

In all cases, the total sterol content lay above the minimum limit (1000 mg/kg) established for virgin olive oils (VOO) [12,13,14], resulting in a mean value of 1490 mg/kg within a range of 1158 to 1943 mg/kg. As expected, the average individual sterol profile obtained in the olive oil from selected Empeltre clones was: *β*-sitosterol as the main sterol (85.58 ± 1.54%), followed by minor contents of Δ5-avenasterol (5.62 ± 1.32%), campesterol (3.16 ± 0.21%), and stigmasterol (1.34 ± 0.81%). The latter sterol displayed a high standard deviation, due to wide variation among the crop years in this study. These results were similar to those which have previously been reported on the Empeltre cultivar [16,22,23,25] and on other Spanish [20,32], Italian [31], Tunisian [35,45], and Algerian [46] cultivars. The percentages of these four sterols enable us to differentiate Empeltre olive oil as a monovarietal oil featuring medium-range contents of *β*-sitosterol and Δ5-avenasterol, but high levels of campesterol and stigmasterol, according to the classification elaborated by Kyçyk et al. [33] on the basis of 43 monovarietal oils from the Córdoba Germplasm Bank.

Lower quantities of cholesterol, 24-methylene-cholesterol, campestanol, clerosterol, sitostanol, Δ5,24-stigmastadienol, Δ7-stigmastenol, and Δ7-avenasterol were found in all samples. On the other hand, brassicasterol, a sterol marker for olive oil adulteration with Brassicaceae oils (e.g., rapeseed, canola), was not detected in any of the samples. Other sterols, such as Δ7-campestanol (present in sunflower oil) and Δ5,23-stigmastadienol (present in refined olive oils due to the refining process) were found in trace amounts in some of the samples (data not shown).

Regarding compliance with the limits established by European [12] and international regulations [13,14] regarding relative sterol composition, not all samples satisfied the requirements to be considered as genuine virgin olive oils (values in bold type in Table 1). The mean percentage of apparent *β*-sitosterol (93.52 ± 0.72%) was above the legal limit [12,13,14] (≥93%): the range of values obtained for this parameter (92.04–94.54%) showed that 26% of all samples analyzed during the three study crop years were non-compliant virgin olive oils. This non-compliance could suggest the presence of seed oils. Kyçyk et al. [33] reported that 23% of the monovarietal oils they analyzed had apparent *β*-sitosterol below 93%. Similarly, Rivera del Álamo et al. [20] described a non-compliance of 15–20% of the commercial Cornicabra virgin olive oils (334 samples) they analyzed during five consecutive crop years. Other authors [28,46,47] have also described monovarietal virgin olive oils from different countries that do not fulfill legislative limits for this parameter.

The mean percentage of Δ7-stigmastenol obtained from the 78 samples of virgin olive oils analyzed in our study was remarkably high (0.46 ± 0.10%), featuring a wide range of values (0.23–0.76%). A total of 12% of the samples had Δ7-stigmastenol values showing above the established 0.5% limit [12,13,14]. Elevated Δ7-stigmastenol values in Empeltre oils had also been observed in studies conducted over the period of 1998 to 2022 [23,24]. Garcia [21,22] has suggested that high Δ7-stigmastenol contents in Empeltre olive oils are a varietal peculiarity. Similar arguments have been propounded by Salvador et al. [18] and Rivera et al. [20] regarding the high campesterol content observed in the Spanish Cornicabra cultivar. High Δ7-stigmastenol content in other monovarietal oils has been described in Argentinian [16], Tunisian [45,46], and Palestinian [48] olive oils. Elevated Δ7-stigmastenol values (%) have likewise been observed in Compositae oils (e.g., sunflower, safflower).

The other sterols all complied with the limits specified in standard regulations for virgin olive oils [12,13,14].

### 3.2. Factors Exerting an Influence on Sterol Composition

The effects of clone type, crop year, and fruit maturity on sterol content and composition are shown in Table 2. Results of three-way ANOVA demonstrated that crop year was the variable under study which most significantly affected [19,49] the majority of the sterols we analyzed, along with total sterol content. Moreover, its effect was statistically significant for all evaluated parameters. Ripening also significantly modulated sterol composition [18,26,28,31,47,49]. Its impact, however, was much less pronounced than that of crop year, except in the case of sitostanol. In contrast, the degree of fruit ripeness was neither significant for Δ7-sterols such as Δ7-stigmastenol and Δ7-avenasterol, nor for clerosterol. Few sterols showed significant differences according to clone. Among them, campesterol and Δ7-stigmastenol were the ones most affected by clone type, and the latter was the factor which exerted the main effect on those sterols. Interactions among two effects (crop year × clone, crop year × fruit maturity, or clone × fruit maturity) showed significant differences for some parameters, but were less notable than independent effects, except for the influence of ripening factor on Δ7-stigmastenol and Δ7-avenasterol. The ripening effect was exclusively significant on Δ7-stigmastenol when interacting with crop year (*p* < 0.001) or clone type (*p* < 0.01).

### 3.3. Changes in Sterol Composition According to Degree of Olive Ripeness

As shown in Table 2, total sterol concentration displayed significant differences according to the olives’ degree of ripeness. In the three crop years studied, no consistent pattern was observed in the evolution of sterol content (Table 3, Table 4, Table 5, Table 6 and Table 7), as similarly observed by Salvador et al. [18] in studies on Cornicabra oils over four consecutive seasons. An evident increase in the concentration of olive oil sterols as the fruit matured (higher than 20%) occurred in 2019. In contrast, most clones showed similar contents in December 2018 compared to the beginning of the sampling, although with some oscillations during the sampling period, except for Clone 2, in which sterols increased by 12%. In 2017, a significant increase in sterols (37%) was observed in December compared to October in olive oils from the “Std” clone, but such variation was minimal or non-existent in the other clones. Other authors have observed a decrease in sterol content [6,32,38] and have explained it as a dilution effect of increasing oil content during fruit ripening [38]. The evolution of sterols directly in the olive pulp [50] has been less studied, but increases in sterol concentration have indeed been observed in the pulp as ripening progresses. Inês et al. [51] suggested that the increase they observed in sterol biosynthesis in early fruits was because the rates of mitosis and membrane formation were at maximum levels. The complexity of the enzymatic pathways involved in sterol biosynthesis [52,53] and the participation of sterols in the biosynthesis of other compounds [37,54], as well as abiotic stress effects on olives (a less studied factor) [53,55], generate a wide range of variability in sterol concentration throughout ripening. Navas-López et al. [30] observed that cultivar type was the main contributor to total variability in sterols. Their study involved seven cultivars grown in five different environments. However, hardly any references have been published [56] regarding the trends of total and individual sterol content during the olive ripening process specifically in the Empeltre cultivar. The dynamics of biosynthesis in the fruit and the accumulation of sterols in Empeltre olive oil could differ from those hitherto described in other cultivars [56].

**Figure 2:**
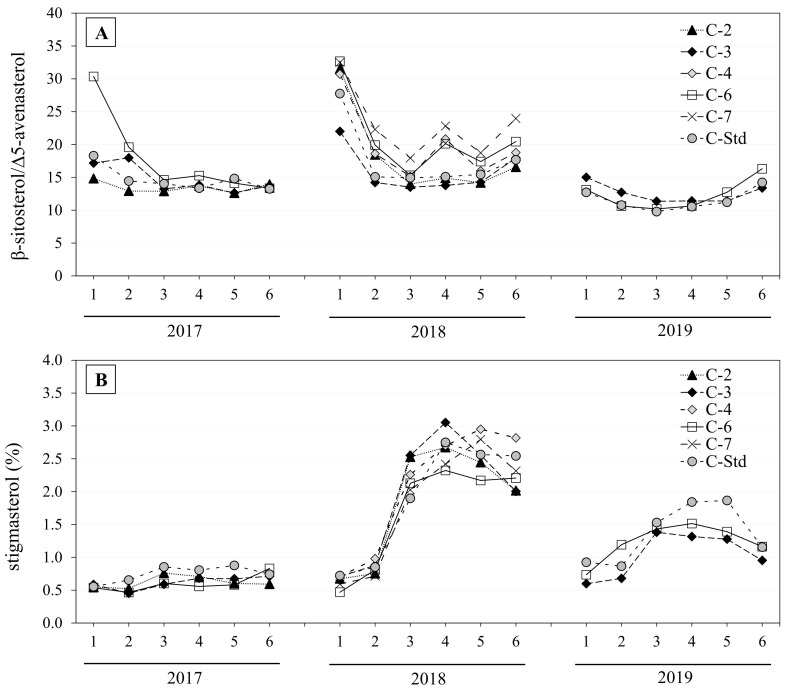
Evolution of the ratio of *β*-sitosterol/Δ5-avenasterol (**A**) and stigmasterol (**B**) by clone (C-2, C-3, C-4, C-6, C-7 and C-Std) throughout sampling in the 2017, 2018, and 2019 crop years. Harvest dates: 1 (1–4 October), 2 (16–18 October), 3 (28 October–7 November), 4 (11–22 November), 5 (25–29 November) and 6 (11–13 December).

In terms of sterol composition, most of the sterols we analyzed presented significant differences according to olive maturity. The evolution of each individual sterol was highly variable (Table 3, Table 4, Table 5, Table 6 and Table 7), but coincided with the most widely observed patterns collected and discussed by Lukić et al. [56] in several monovarietal oils. In general, in our study, the net change in relative *β*-sitosterol content was slightly negative at the end of the sampling period (December). During the initial sampling period (October and early November), initial *β*-sitosterol content decreased and stabilized until mid-December, the last sampling date. In the 2019 harvest, this behavior differed in “Std” and Clone 6, as *β*-sitosterol relative levels increased in those two clone types in December. The changes we observed in the *β*-sitosterol pattern during ripening suggest a modification of the biosynthesis of this sterol or of oil accumulation in the fruit, as its stabilization coincides with the end of the lipogenesis period [56]. Meanwhile, Δ5-avenasterol showed a trend that was inverse to *β*-sitosterol, as indicated by the strong negative correlation obtained among those two sterols (r = −0.902, *p* < 0.01), likewise observed in other monovarietal oils from several countries [6,32,57,58]. In our study, Δ5-avenasterol increased by 30% on average during the sampling period, but its evolution was different in 2019, as occurred with *β*-sitosterol. During that year, a decrease in initial Δ5-avenasterol content was predominant, especially in the oils coming from Clone 6. In line with this, Gutiérrez et al. [38] have already hypothesized that the enzymatic activity that regulates the *β*-sitosterol/Δ5-avenasterol ratio was the cause of the high correlation they observed in their study. Indeed, Δ5-avenasterol is the precursor in the biosynthesis of *β*-sitosterol [52,59]. Several other authors [26,60] indicate that the *β*-sitosterol/Δ5-avenasterol ratio can decrease as fruit ripening progresses due to the decrease in the proportion of oil to stone, which is rich in *β*-sitosterol, but poor in Δ5-avenasterol. Fernández-Cuesta et al. [50] have ruled out the latter hypothesis by observing significant changes in both sterols directly in the pulp as the fruit ripened, thereby confirming that the activity of the enzymes involved in the biosynthesis of both sterols was responsible for the differences in their contents. This hypothesis would explain the high negative correlation we found between the two compounds. Other studies have found no significant differences between oils obtained from whole fruit versus oils obtained exclusively from olive pulp [26,60]. Figure 2A shows the evolution of the *β*-sitosterol/Δ5-avenasterol ratio in the olive oils obtained in our investigation over the three years of study.

Relative campesterol content decreased slightly during sampling [47,61] (Table 3, Table 4, Table 5, Table 6 and Table 7). Although this was the prevalent trend, a relative uniformity was likewise observed [29,38,61], especially in the 2018 crop year. As for stigmasterol, most of the studies reported in the review by Lukić et al. [56] describe an increase in its content, especially in the later stages of ripening, although that increase is not significant (as opposed to our results). However, the study of the effect of ripening on stigmasterol content presented difficulties in our trial. This sterol’s increase varied considerably, depending on the crop year (Figure 2B), which caused a high dispersion in our set of results. The highest average increases observed during the overall sampling period were in 2018 (72%) and 2019 (31%). Intense increases in late October-early November were observed during 2018 and 2019, after which they stabilized or slightly decreased in December. The increase in stigmasterol was so pronounced in mid-autumn 2018 that it implied regulatory non-compliance for the parameter for apparent *β*-sitosterol [12,13,14] throughout that sampling period (values in bold in Table 3, Table 4, Table 5, Table 6 and Table 7). The corresponding increment was lower in the crop year 2017 (22%). The possible relation we observed between stigmasterol and apparent *β*-sitosterol was confirmed by their high negative correlation (r = −0.975; *p* < 0.01). This association has previously been reported in Empeltre [21] and other cultivars [29,33,35]. In contrast, there was no significant correlation with *β*-sitosterol, although it is the precursor in the biosynthesis of stigmasterol [52,59]. Certain authors have related the stigmasterol content in olive oil with fruit quality and therefore, with olive oil quality, confirming a positive correlation between high acidity and the amount of stigmasterol [21,62]. No such relationship was found in our investigation, as the olive oils we studied had very low acidity (Table 1), which indicated satisfactory olive health.

No correlation was observed between fruit ripening and the parameter for apparent *β*-sitosterol.

The effect of ripeness on the relative amounts of minority sterols was varied. Cholesterol remained relatively constant, but 24-methylene-cholesterol, an intermediate metabolite in the synthesis of campesterol, increased significantly. Other minority sterols, such as Δ7-stigmastenol and Δ7-avenasterol, were not affected by fruit ripening (Table 2), but showed significant differences in terms of crop year × maturity interaction. Those two Δ7-sterols, which correlated positively with each other [37] (r = 0.793, *p* < 0.01), displayed parallel trends throughout ripening, according to the year under study (upward in 2017, downward in 2018, and relatively constant in 2019). Sitostanol, one of the main stanols in olive oil (a hydrogenated form of *β*-sitosterol, its precursor), decreased drastically throughout the sampling. This sterol was the only one which correlated significantly with the ripening index (r = −0.866; *p* < 0.01). Sakouhi et al. [37] ascribed the decrease in the stanols sitostanol and campestanol to their conversion into brassinosteroids, i.e., steroid hormones that regulate plant growth and development.

### 3.4. Changes in Sterol Composition by Crop Year Effect

Although our study did not directly address environmental factors, such as temperature, solar radiation (UV), precipitation, or humidity, these, among other aspects, could have been responsible for the significant interannual differences observed in sterol composition. Recent studies reported by Du et al. [53] indicate that enzymes involved in the sterol biosynthesis pathway are key in plant responses to abiotic stress. Drought is the most persistent and therefore, the most studied abiotic stress in olives, although high temperatures and UV-B radiation activate adaptive mechanisms that are not well known [55]. Such environmental factors have been scarcely addressed directly in relation to the modulation of sterol composition in olive fruit, and thus also in olive oil, although their influence has been demonstrated [26,55]. Many further studies have evidenced the effect of the environment in trials conducted at geographical locations with different climatic characteristics [24,27,28,34,35,48].

The oils from crop year 2017 had the highest sterol concentration and the highest relative contents of Δ7-stigmastenol and Δ7-avenasterol, but the lowest contents of stigmasterol. The 2017 autumn harvest was characterized by extreme drought (16 mm of autumn rainfall only), combined with the widest thermal oscillation and the lowest minimum temperatures (Figure 1): all these factors caused a substantial loss of moisture in the fruit [63]. In contrast, the autumn of 2018 was extremely rainy (10 times more rainfall than in autumn 2017) and had the narrowest thermal oscillation. Immediately after the high precipitation period in October 2018, olive oils had the highest stigmasterol amounts, but lower percentages of Δ5-avenasterol and apparent *β*-sitosterol (resulting in non-compliant samples; values in bold in Table 3, Table 4, Table 5, Table 6 and Table 7). Among the three crop years we studied, this circumstance only occurred in 2018. The lowest relative amounts of *β*-sitosterol, but the highest of Δ5-avenasterol, were observed in the 2019 season. Summer and autumn of 2019 had higher average and maximum temperatures, although these differences were not pronounced compared to the conditions in 2018.

**Figure 3:**
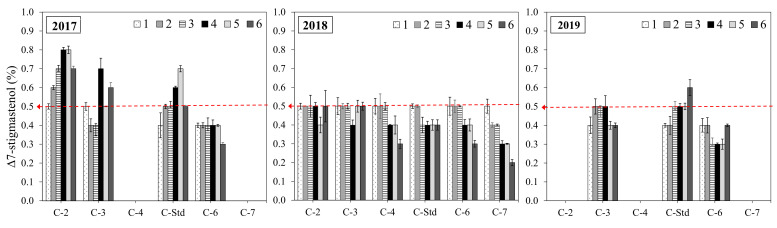
Evolution of Δ7-stigmastenol (%) according to harvest date and clone (C-2, C-3, C-4, C-6, C-7, and C-Std) in the three crop years studied (2017, 2018, and 2019). Harvest dates: 1 (1–4 October), 2 (16–18 October), 3 (28 October–7 November), 4 (11–22 November), 5 (25–29 November), and 6 (11–13 December). Non-compliance norm: Δ7-stigmastenol > 0.05 (%). Red arrow: limit established by the current EU/IOC/CODEX regulatory for OOV for Δ7-stigmastenol ≤ 0.5%.

In line with these results, several authors have likewise described an increase in stigmasterol associated with greater water availability in Empeltre [24] and other cultivars [64,65]; however, Guillaume et al. [26] described lower stigmasterol and apparent *β*-sitosterol in association with higher levels of irrigation in Australian oils. On the other hand, higher contents of Δ7-stigmastenol and total sterols have been described in regions or seasons with low rainfall, or even in studies featuring irrigation deficit in Empeltre [21] and other cultivars [26,27,64], in line with our results. Figure 3 clearly shows the influence of crop year on compliance with the regulatory limit for Δ7-stigmastenol (0.5%). A variation in sterol content associated with water treatments is not evident; a significant decrease in sterol concentration has been described [64,65], but inconsistency can also be noted in results [66] stemming from irrigated cultivars.

The direct influence of temperature on sterol composition in olive fruit and olive oils has been less studied. Hamze et al. [36] conducted temperature-controlled experiments using open chambers. Their experiments reported an increase in sterol concentration caused by a moderate increment in air temperature (3–4 °C), in contrast with our study. That increment also induced an increase in stigmasterol, whereas apparent *β*-sitosterol and Δ5-avenasterol decreased. Those results are in line with Piravi-Vanak et al. [34], who reported that cold climate regions generate oils with higher *β*-sitosterol and lower stigmasterol content.

### 3.5. Changes in Sterol Composition According to Clonal Type

No significant differences were observed for most sterols in regard to clone type (Table 2). Δ7-stigmastenol, and especially campesterol, were the most affected sterols among those which showed significant differences. Along with total sterol concentration, Δ7-avenasterol and Δ5,24-stigmastadienol displayed significant differences, but with low effect. Results from Arbequina [24,67] and Empeltre [46,67] clonal selections yielded significant differences in very few parameters caused by clone type in olive fruit and/or olive oil.

Several studies have reported a considerable genetic influence on campesterol content [20,26,29,32], thereby corroborating the pronounced influence of clone type on this sterol in olive oils. In our study, Clone 6 presented higher values in this respect than the other clones.

Figure 3 shows the effects of clone type on Δ7-stigmastenol. Most Δ7-stigmastenol values were generally high; the “Std” Clone and Clone 2 had the highest rate of non-compliances, and most of these could be observed in 2017. In contrast, Clone 6 displayed the lowest values for this sterol. This clonal characteristic should be taken into account by producers who want to select clones for olive oils whose authenticity is not called into question.

### 3.6. Discriminant Analysis

**Figure 4:**
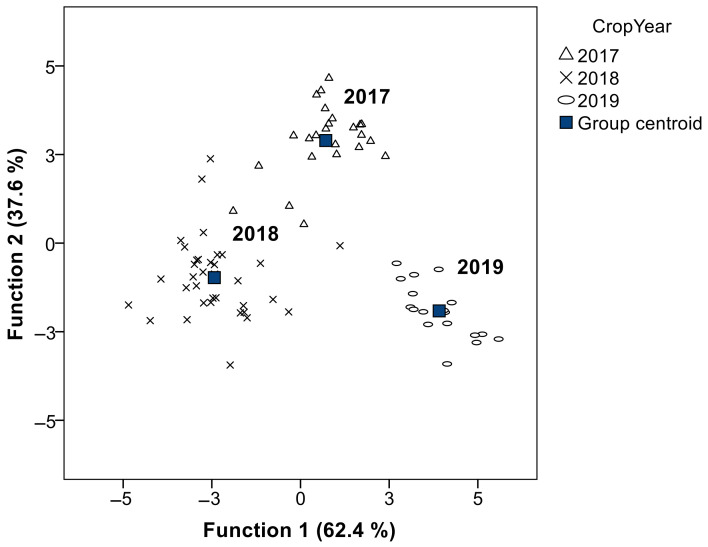
Canonical discriminant functions biplot and centroids of crop years obtained from sterols composition.

We applied a canonical discriminant analysis to visualize the discrimination capacity of the sterol composition of the Empeltre olive oils under study. The grouping variable “crop year” was the one displaying the best classifying ability (94.9% of the oils were classified correctly). The six most discriminating variables were total sterols, Δ5,24-stigmastadienol, Δ7-avenasterol, Δ5-avenasterol, stigmasterol, and campesterol. Figure 4 shows the 78 samples projected onto a biplot defined by the two canonical discriminant functions that explained 100% of the total variation (*p* < 0.001). The classification results (Table 8) show an effective separation between the three years under study based on sterol composition. The year 2017 showed greater differentiation compared to the other two years. According to Canonical Discriminant Function 1 (63.9% of the total variance), the 2018 samples were differentiated by higher b-sitosterol and campesterol contents, but lower Δ5-avenasterol compared to 2019. Canonical Discriminant Function 2 (36.1% of the total variance) differentiated the years 2018 and 2019 versus 2017 by higher stigmasterol but lower total sterols, apparent *β*-sitosterol, and Δ7-stigmasternol.

## 4. Conclusions

The results presented in this study regarding the characterization of sterol composition in Empeltre olive oils have revealed high percentages of non−compliances vis−à−vis official regulations that determine an olive oil’s presumed authenticity. In general, sterols were mainly affected by harvest year and less by degree of ripening. The least significant effect was clone type, although this latter factor exerted the most pronounced effect on campesterol and Δ7−stigmasterol. The high Δ7−stigmastenol content observed in the Empeltre olive oils in our study reinforces the hypothesis regarding varietal peculiarity raised previously. However, the non−compliances we detected in this sterol could be caused by particular environmental factors associated with crop year, as revealed by the results of canonical discriminant analysis (DA). Environmental factors could also be responsible for the non−compliance rate of apparent *β*−sitosterol, which is affected by an increase in stigmasterol content.

Our results suggest that commercial standards should generally take natural variations in sterol composition into account with the purpose of avoiding economic damage to certain producers whose single−varietal olive oils have abnormal sterol content, while still ensuring that consumers are protected from fraud.

## Figures and Tables

**Table 1:** Descriptive statistic of physicochemical quality parameters and sterol composition during three crop years (2017, 2018, and 2019) (*n* = 78).

Parameter	Mean ± sd	Range	Percentiles			
			25	50	75	90
free acidity (% oleic acid)	0.16 ± 0.04	0.07–0.26	0.13	0.15	0.18	0.13
peroxides value (meq O_2_/kg)	6.3 ± 2.7	2.2–14.5	4.28	5.45	8.03	4.28
K_270_	0.11 ± 0.02	0.07–0.17	0.10	0.11	0.12	0.10
K_232_	1.67 ± 0.17	1.38–2.08	1.55	1.64	1.79	1.55
Cholesterol ^a^	0.08 ± 0.02	0.05–0.15	0.07	0.08	0.10	0.11
Brassicasterol ^a^	nd	nd	nd	nd	nd	nd
24-methylene-cholesterol	0.07 ± 0.04	0.01–0.22	0.05	0.06	0.09	0.12
Campesterol ^a^	3.16 ± 0.21	2.77–3.59	2.98	3.17	3.31	3.45
campestanol	0.38 ± 0.05	0.30–0.57	0.34	0.37	0.42	0.45
Stigmasterol ^a^	1.34 ± 0.81	0.45–3.05	0.68	0.90	2.14	2.58
clerosterol	1.02 ± 0.13	0.49–1.45	0.95	1.00	1.07	1.17
β-sitosterol	85.58 ± 1.54	81.87–89.36	84.70	85.63	86.39	87.66
sitostanol	0.47 ± 0.12	0.30–0.85	0.38	0.44	0.56	0.65
Δ5-avenasterol	5.62 ± 1.32	2.74–8.37	4.74	5.80	6.45	7.37
Δ5,24-stigmastadienol	0.82 ± 0.20	0.47–1.29	0.66	0.78	1.00	1.12
Δ7-stigmastenol ^a^	0.46 ± 0.10	0.23–0.76	0.40	0.45	0.51	0.60
Δ7-avenasterol	0.95 ± 0.22	0.47–1.47	0.79	0.96	1.09	1.25
app. β-sitosterol ^a^	93.52 ± 0.72	92.04–94.54	92.86	93.79	94.13	94.26
total sterols ^a^	1490 ± 204	1158–1943	1312	1452	1696	1792

*^a^* Limits established by the current EU/IOC/CODEX regulatory for OOV: total sterols ≥ 1000 mg/kg; cholesterol ≤ 0.5%; brassicasterol ≤ 0.1%; campesterol ≤ 4.0%; stigmasterol ≤ campesterol; Δ7-stigmastenol ≤ 0.5%; app. *β*-sitosterol ≥ 93.0%. Values in bold: non-compliant. Not detected = nd. App. *β*-sitosterol = clerosterol + *β*-sitosterol + sitostanol + Δ5-avenasterol + Δ5,24-stigmastadienol.

**Table 2:** Analysis of variance (F-values) by 3-way ANOVA of sterol composition in three crop years (Y) (2017, 2018, and 2019) on four Empeltre clones (C) (2, 3, 6, and Std) and in three fruit maturity stages (M) (green, spotted, and ripe).

Parameter	Crop Year (Y)	Clone (C)	Fruit Maturity (M)	Y × C	Y × M	C × M	Y × C × M
cholesterol *^a^*	34.8 ***	0.8	6.3 **	3.0 *	1.4	1.4	2.1
24-methylene-cholesterol	26.5 ***	4.6 **	19.9 ***	2.9 *	0.9	1.3	1.2
campesterol *^a^*	11.2 ***	41.8 ***	13.9 ***	7.3 ***	3.5 *	3.2 **	1.1
campestanol	6.2 **	4.3 **	4.7 *	2.6*	0.7	1.3	0.8
stigmasterol *^a^*	37.8 ***	0.5	8.3 ***	0.2	2.7	0.4	0.3
clerosterol	8.1 ***	1.8	0.9	1.7	1.6	1.7	2.3 *
*β*-sitosterol	14.3 ***	2.2	8.6 ***	1.4	0.9	0.5	0.5
sitostanol	5.7 **	2	25.7 ***	2.2	0.3	0.1	0.3
Δ5-avenasterol	24.9 ***	2.1	7.6 **	1.3	1.9	0.2	0.7
Δ5,24-stigmastadienol	72.8 ***	8.2 ***	3.5 *	3.8 **	4.8 **	2	1.2
Δ7-stigmastenol *^a^*	15.1 ***	20.4 ***	1.8	3.5 **	6 ***	3.8 **	0.9
Δ7-avenasterol	31.8 ***	17 ***	3	3.1 *	6.8 ***	1.8	0.8
app. *β*-sitosterol *^a^*	27.6 ***	1.1	7.4 **	0.4	1	0.6	0.5
*β*-sitosterol/Δ5-avenasterol	7.6 **	1.3	3.8 *	0.5	0.7	0.1	0.4
total sterols *^a^*	54.8 ***	5.9 **	13.3 ***	4.2 **	1.3	1.0	2.0

Significance levels: *, *p* < 0.05; **, *p* < 0.01; ***, *p* < 0.001. Absence of *: no significant differences. *^a^* Parameters regulated by EU regulation and IOC/CODEX standards. Interactions: between crop year and clone (Y × C), between crop year and fruit maturity (Y × M), between clone and fruit maturity (C × M), between crop year, clone, and fruit maturity (Y × C × M).

**Table 3:** Evolution of sterol composition (%) and total sterols (mg/kg) according to picking date ^a^ for clone 2 in the crop years 2017, 2018, and 2019.

	Clone 2 (C-2)											
	Crop year 2017						Crop year 2018					
Parameter	4 Oct	18 Oct	7 Nov	22 Nov	29 Nov	13 Dec	1 Oct	15 Oct	28 Oct	11 Nov	25 Nov	11 Dec
ripening index	2.4	3.2	4.0	5.1	5.5	6.1	1.1	1.7	2.7	2.9	3.6	4.2
cholesterol ^b^	0.05 ± 0.01a	0.06 ± 0.00a	0.07 ± 0.01a	0.06 ± 0.00a	0.06 ± 0.01a	0.06 ± 0.00a	0.08 ± 0.01a	0.11 ± 0.01bc	0.10 ± 0.00ab	0.10 ± 0.00abc	0.12 ± 0.01c	0.12 ± 0.00c
brassicasterol ^b^	nd	nd	nd	nd	nd	nd	nd	nd	nd	nd	nd	nd
24-methylene-cholesterol	0.04 ± 0.01a	0.05 ± 0.01a	0.06 ± 0.01a	0.07 ± 0.01a	0.06 ± 0.00a	0.07 ± 0.01a	0.02 ± 0.00a	0.04 ± 0.00a	0.06 ± 0.00b	0.05 ± 0.02b	0.07 ± 0.01b	0.06 ± 0.02b
campesterol ^b^	2.99 ± 0.02a	2.83 ± 0.02a	2.86 ± 0.00a	2.86 ± 0.06a	2.81 ± 0.02a	2.84 ± 0.06a	3.33 ± 0.05a	3.21 ± 0.03b	3.10 ± 0.03c	3.10 ± 0.05c	3.07 ± 0.01c	3.14 ± 0.00bc
campestanol	0.34 ± 0.03a	0.32 ± 0.02a	0.35 ± 0.01a	0.30 ± 0.03a	0.32 ± 0.01a	0.30 ± 0.00a	0.41 ± 0.04a	0.46 ± 0.00a	0.41 ± 0.11a	0.31 ± 0.03a	0.37 ± 0.03a	0.34 ± 0.02a
stigmasterol ^b^	0.54 ± 0.01a	0.53 ± 0.00b	0.76 ± 0.00c	0.71 ± 0.01d	0.60 ± 0.01e	0.59 ± 0.01e	0.67 ± 0.00a	0.75 ± 0.02b	2.53 ± 0.02c	2.67 ± 0.01d	2.45 ± 0.01e	2.02 ± 0.01f
clerosterol	1.02 ± 0.03a	0.99 ± 0.00a	0.92 ± 0.04b	0.93 ± 0.00b	0.93 ± 0.00b	0.92 ± 0.01b	1.13 ± 0.01a	0.99 ± 0.03a	nd	0.95 ± 0.08a	1.00 ± 0.00a	0.94 ± 0.03a
β-sitosterol	86.00 ± 0.04a	85.13 ± 0.17bc	84.74 ± 0.22b	85.31 ± 0.18c	85.00 ± 0.21bc	86.00 ± 0.09a	88.46 ± 0.14a	86.57 ± 0.01b	83.82 ± 0.21c	84.38 ± 0.07d	84.65 ± 0.10d	85.97 ± 0.03e
sitostanol	0.47 ± 0.02a	0.41 ± 0.01ab	0.37 ± 0.03bc	0.32 ± 0.05c	0.32 ± 0.02c	0.31 ± 0.02c	0.85 ± 0.06a	0.59 ± 0.02b	0.48 ± 0.00c	0.46 ± 0.01c	0.39 ± 0.03d	0.36 ± 0.02d
Δ5-avenasterol	5.80 ± 0.05a	6.59 ± 0.05b	6.57 ± 0.18b	6.23 ± 0.12c	6.74 ± 0.17b	6.16 ± 0.10c	2.81 ± 0.11a	4.69 ± 0.04b	5.99 ± 0.11c	5.67 ± 0.12d	5.97 ± 0.04c	5.19 ± 0.05e
Δ5,24-stigmastadienol	0.93 ± 0.03a	1.06 ± 0.04b	1.13 ± 0.01c	1.04 ± 0.04b	1.01 ± 0.01b	0.90 ± 0.01a	0.78 ± 0.05ab	0.88 ± 0.04a	0.89 ± 0.04a	0.77 ± 0.10ab	0.66 ± 0.00bc	0.61 ± 0.00c
Δ7-stigmastenol ^b^	0.53 ± 0.01a	**0.56 ± 0.01a**	**0.69 ± 0.02b**	**0.76 ± 0.01c**	**0.74 ± 0.02c**	**0.67 ± 0.01b**	0.52 ± 0.02a	0.54 ± 0.00a	0.48 ± 0.06a	0.46 ± 0.02a	0.40 ± 0.04a	0.45 ± 0.08a
Δ7-avenasterol	1.29 ± 0.02a	1.39 ± 0.01b	1.47 ± 0.00b	1.42 ± 0.06b	1.42 ± 0.03b	1.17 ± 0.04c	0.89 ± 0.01a	1.12 ± 0.03b	1.09 ± 0.03b	1.01 ± 0.02c	0.81 ± 0.00d	0.80 ± 0.03d
app. β-sitosterol ^b^	94.22 ± 0.11a	94.18 ± 0.14ab	93.73 ± 0.02c	93.82 ± 0.02cd	94.00 ± 0.01bd	94.30 ± 0.03a	94.02 ± 0.09a	93.73 ± 0.00b	**92.18 ± 0.08c**	**92.23 ± 0.07c**	**92.67 ± 0.09d**	93.07 ± 0.09e
total sterols ^b^	1807 ± 6a	1791 ± 18a	1796 ± 43a	1943 ± 44b	1860 ± 28ac	1893 ± 27bc	1381 ± 10a	1275 ± 21a	1303 ± 227a	1470 ± 43a	1448 ± 0a	1567 ± 19a

Values are mean ± standard deviation (*n* = 2). ^a^ Different letters for each parameter indicate significant statistical differences (*p* < 0.05) among picking dates for each crop year (a–f). ^b^ Limits established by the current regulation: total sterols ≥1000 mg/kg; cholesterol ≤ 0.5%; brassicasterol ≤ 0.1%; campesterol ≤ 4.0%; stigmasterol ≤ campesterol; Δ7-stigmastenol ≤ 0.5%; app. β-sitosterol ≥ 93.0%. Values in bold: non-compliance. Not detected = nd.

**Table 4:** Evolution of sterol composition (%) and total sterols (mg/kg) according to picking date ^a^ for clone 3 in the crop years 2017, 2018, and 2019.

	Clone 3 (C-3)																	
	Crop year 2017						Crop year 2018						Crop year 2019					
Parameter	4 Oct	18 Oct	7 Nov	22 Nov	29 Nov	13 Dec	1 Oct	15 Oct	28 Oct	11 Nov	25 Nov	11 Dec	4 Oct	18 Oct	7 Nov	22 Nov	29 Nov	13 Dec
ripening index	1.7	2.0	3.5	4.9	4.9	5.3	1.5	2.7	3.3	3.9	4.4	4.8	1.9	3.1	3.2	4.1	3.9	5.3
cholesterol ^b^	0.05 ± 0.00a	0.06 ± 0.01a	0.06 ± 0.01a	0.06 ± 0.01a	0.06 ± 0.01a	0.07 ± 0.02a	0.09 ± 0.01a	0.09 ± 0.01a	0.09 ± 0.00a	0.08 ± 0.01a	0.11 ± 0.01a	0.10 ± 0.00a	0.07 ± 0.01a	0.11 ± 0.01bc	0.12 ± 0.03c	0.07 ± 0.00a	0.09 ± 0.01abc	0.08 ± 0.01ab
brassicasterol ^b^	nd	nd	nd	nd	nd	nd	nd	nd	nd	nd	nd	nd	nd	nd	nd	nd	nd	nd
24-methylene-cholesterol	0.03 ± 0.00a	0.04 ± 0.00ab	0.06 ± 0.01c	0.05 ± 0.0bc	0.07 ± 0.01c	0.06 ± 0.01bc	0.03 ± 0.00a	0.05 ± 0.00bca	0.06 ± 0.01bcd	0.04 ± 0.02ab	0.08 ± 0.01d	0.07 ± 0.01cd	0.04 ± 0.01a	0.08 ± 0.01ab	0.11 ± 0.01bc	0.11 ± 0.02bc	0.13 ± 0.00c	0.10 ± 0.04bc
campesterol ^b^	3.18 ± 0.00a	3.23 ± 0.01b	3.26 ± 0.01c	2.93 ± 0.02d	3.01 ± 0.00e	2.93 ± 0.00d	3.27 ± 0.03a	3.08 ± 0.00b	2.98 ± 0.03c	2.95 ± 0.06c	2.86 ± 0.02d	3.03 ± 0.03bc	3.22 ± 0.01a	2.97 ± 0.01bc	2.92 ± 0.04bc	2.88 ± 0.05b	2.99 ± 0.03c	2.93 ± 0.05bc
campestanol	0.31 ± 0.01a	0.34 ± 0.01b	0.35 ± 0.02b	0.34 ± 0.01b	0.35 ± 0.01b	0.30 ± 0.01a	0.40 ± 0.01a	0.37 ± 0.00a	0.40 ± 0.04a	0.35 ± 0.05a	0.38 ± 0.00a	0.38 ± 0.01a	0.35 ± 0.01a	0.34 ± 0.02a	0.34 ± 0.03a	0.37 ± 0.08a	0.40 ± 0.04a	0.36 ± 0.05a
stigmasterol ^b^	0.59 ± 0.01a	0.45 ± 0.00b	0.59 ± 0.01a	0.68 ± 0.01c	0.67 ± 0.00c	0.71 ± 0.00d	0.71 ± 0.02a	0.88 ± 0.01b	2.55 ± 0.02c	3.05 ± 0.05d	2.56 ± 0.01c	2.00 ± 0.02e	0.60 ± 0.02a	0.68 ± 0.02a	1.38 ± 0.01b	1.32 ± 0.15b	1.28 ± 0.02b	0.95 ± 0.01c
clerosterol	1.04 ± 0.00a	1.05 ± 0.01a	0.98 ± 0.03b	0.93 ± 0.01c	0.93 ± 0.01c	0.90 ± 0.01c	1.03 ± 0.02a	1.01 ± 0.01a	0.98 ± 0.06a	0.95 ± 0.02a	0.90 ± 0.04a	0.96 ± 0.07a	1.04 ± 0.04a	1.02 ± 0.07a	0.99 ± 0.05a	1.09 ± 0.19a	1.24 ± 0.16a	1.25 ± 0.23a
β-sitosterol	86.92 ± 0.10a	87.26 ± 0.06b	85.44 ± 0.15c	85.35 ± 0.03cd	85.16 ± 0.06d	85.82 ± 0.03e	87.66 ± 0.14a	85.92 ± 0.66b	83.75 ± 0.22c	83.81 ± 0.01c	84.70 ± 0.21d	86.37 ± 0.01b	86.42 ± 0.39a	84.82 ± 0.26b	83.71 ± 0.14c	83.66 ± 0.80c	83.70 ± 0.10c	85.28 ± 0.27b
sitostanol	0.60 ± 0.03a	0.63 ± 0.05a	0.56 ± 0.04a	0.38 ± 0.01b	0.39 ± 0.01b	0.38 ± 0.00b	0.64 ± 0.02a	0.44 ± 0.00b	0.41 ± 0.03bc	0.38 ± 0.02ce	0.36 ± 0.01e	0.34 ± 0.07e	0.55 ± 0.04a	0.51 ± 0.08a	0.37 ± 0.02b	0.37 ± 0.04b	0.33 ± 0.01b	0.32 ± 0.03b
Δ5-avenasterol	5.06 ± 0.09a	4.85 ± 0.06b	6.45 ± 0.01c	6.18 ± 0.03d	6.74 ± 0.06e	6.26 ± 0.01d	3.99 ± 0.06a	6.04 ± 0.01bd	6.20 ± 0.04c	6.08 ± 0.06bc	5.94 ± 0.05d	4.81 ± 0.01e	5.76 ± 0.12a	6.67 ± 0.08b	7.37 ± 0.08c	7.33 ± 0.23c	7.34 ± 0.13c	6.37 ± 0.02c
Δ5,24-stigmastadienol	0.78 ± 0.03ae	0.68 ± 0.02b	0.70 ± 0.05abe	1.02 ± 0.00c	0.88 ± 0.02d	0.78 ± 0.04e	0.85 ± 0.01a	0.47 ± 0.66a	0.94 ± 0.02a	0.79 ± 0.00a	0.67 ± 0.01a	0.63 ± 0.02a	0.78 ± 0.10a	1.16 ± 0.02a	1.13 ± 0.05a	1.11 ± 0.03a	1.11 ± 0.22a	1.04 ± 0.18a
Δ7-stigmastenol ^b^	0.46 ± 0.02ab	0.45 ± 0.03ab	0.42 ± 0.01a	**0.67 ± 0.06c**	0.52 ± 0.00bd	**0.60 ± 0.03cd**	0.50 ± 0.05a	0.51 ± 0.01a	0.50 ± 0.02a	0.44 ± 0.03a	0.51 ± 0.03a	0.45 ± 0.02a	0.39 ± 0.04a	0.48 ± 0.04a	0.45 ± 0.00a	0.51 ± 0.06a	0.43 ± 0.02a	0.44 ± 0.01a
Δ7-avenasterol	0.99 ± 0.01a	0.96 ± 0.01b	1.13 ± 0.00c	1.41 ± 0.02d	1.23 ± 0.01e	1.19 ± 0.01d	0.84 ± 0.03a	1.09 ± 0.01b	1.06 ± 0.00b	1.00 ± 0.00c	0.93 ± 0.02d	0.71 ± 0.00e	0.78 ± 0.02a	1.12 ± 0.05b	1.05 ± 0.04b	1.14 ± 0.06b	0.96 ± 0.06bc	0.90 ± 0.03c
app. β-sitosterol ^b^	94.40 ± 0.01a	94.47 ± 0.05a	94.13 ± 0.03b	93.86 ± 0.06c	94.09 ± 0.03b	94.14 ± 0.02b	94.16 ± 0.09a	93.90 ± 0.04b	**92.33 ± 0.14c**	**92.04 ± 0.08d**	**92.58 ± 0.10e**	93.16 ± 0.04f	94.54 ± 0.09a	94.17 ± 0.02a	93.57 ± 0.14b	93.56 ± 0.35b	93.72 ± 0.16b	94.25 ± 0.15a
total sterols ^b^	1747 ± 7acd	1699 ± 11bd	1695 ± 11bd	1762 ± 4c	1718 ± 16d	1823 ± 19e	1358 ± 40a	1305 ± 0.09b	1296 ± 15b	1446 ± 0c	1297 ± 13cd	1372 ± 1e	1378 ± 12a	1462 ± 25ab	1516 ± 26b	1477 ± 88ab	1534 ± 42b	1641 ± 7c

Values are mean ± standard deviation (*n* =2). ^a^ Different letters for each parameter indicate significant statistical differences (*p* < 0.05) among picking dates for each crop year (a–f). ^b^ Limits established by the current regulation: total sterols ≥ 1000 mg/kg; cholesterol ≤ 0.5%; brassicasterol ≤ 0.1%; campesterol ≤ 4.0%; stigmasterol ≤ campesterol; Δ7-stigmastenol ≤ 0.5%; app. β-sitosterol ≥ 93.0%. Values in bold: non-compliance. Not detected = nd.

**Table 5:** Evolution of sterol composition (%) and total sterols (mg/kg) according to picking date ^a^ for clone 6 in the crop years 2017, 2018, and 2019.

	Clone 6(C-6)																	
	Crop year 2017						Crop year 2018						Crop year 2019					
Parameter	4 Oct	18 Oct	7 Nov	22 Nov	29 Nov	13 Dec	1 Oct	15 Oct	28 Oct	11 Nov	25 Nov	11 Dec	4 Oct	18 Oct	7 Nov	22 Nov	29 Nov	13 Dec
ripening index	0.6	1.2	3.7	3.9	4.4	5.3	0.8	1.8	2.7	2.9	3.7	3.9	2.0	2.8	3.4	3.9	4.4	5.0
cholesterol ^b^	0.05 ± 0.00a	0.06 ± 0.01a	0.07 ± 0.00ab	0.07 ± 0.01ab	0.08 ± 0.02bc	0.09 ± 0.00c	0.07 ± 0.02a	0.10 ± 0.02a	0.09 ± 0.01a	0.08 ± 0.00a	0.12 ± 0.00a	0.10 ± 0.01a	0.08 ± 0.00a	0.07 ± 0.01a	0.10 ± 0.05a	0.07 ± 0.01a	0.10 ± 0.04a	0.08 ± 0.02a
brassicasterol ^b^	nd	nd	nd	nd	nd	nd	nd	nd	nd	nd	nd	nd	nd	nd	nd	nd	nd	nd
24-methylene-cholesterol	0.01 ± 0.02a	0.04 ± 0.01ab	0.06 ± 0.01ac	0.08 ± 0.02cd	0.08 ± 0.00cd	0.10 ± 0.01d	0.04 ± 0.02ab	0.03 ± 0.00a	0.05 ± 0.01ab	0.05 ± 0.01ab	0.06 ± 0.01b	0.07 ± 0.01b	0.04 ± 0.00a	0.11 ± 0.01b	0.14 ± 0.00c	0.22 ± 0.02d	0.18 ± 0.01e	0.13 ± 0.01bc
campesterol ^b^	3.59 ± 0.04a	3.45 ± 0.03b	3.52 ± 0.00b	3.47 ± 0.03b	3.46 ± 0.01b	3.30 ± 0.02c	3.37 ± 0.11b	3.26 ± 0.02ab	3.14 ± 0.01a	3.33 ± 0.01b	3.27 ± 0.00b	3.33 ± 0.02b	3.30 ± 0.04a	3.20 ± 0.00b	3.13 ± 0.04c	3.29 ± 0.04a	3.28 ± 0.03a	3.31 ± 0.01a
campestanol	0.35 ± 0.01ab	0.37 ± 0.02a	0.33 ± 0.01b	0.35 ± 0.02ab	0.36 ± 0.00ab	0.34 ± 0.00ab	0.37 ± 0.00a	0.36 ± 0.00a	0.40 ± 0.04a	0.36 ± 0.03a	0.44 ± 0.06a	0.32 ± 0.01a	0.43 ± 0.01a	0.45 ± 0.07a	0.44 ± 0.01a	0.57 ± 0.09a	0.45 ± 0.03a	0.36 ± 0.01a
stigmasterol ^b^	0.54 ± 0.01a	0.47 ± 0.01b	0.60 ± 0.00c	0.56 ± 0.03ac	0.58 ± 0.00bc	0.83 ± 0.01d	0.47 ± 0.01a	0.81 ± 0.01b	2.13 ± 0.00c	2.32 ± 0.01d	2.17 ± 0.02e	2.21 ± 0.02e	0.74 ± 0.02a	1.19 ± 0.03b	1.43 ± 0.07cd	1.51 ± 0.06d	1.39 ± 0.01c	1.16 ± 0.03b
clerosterol	1.07 ± 0.01a	1.02 ± 0.01ab	0.99 ± 0.01bc	0.92 ± 0.05d	0.95 ± 0.01cd	0.93 ± 0.00d	1.02 ± 0.01a	1.06 ± 0.00b	1.01 ± 0.01a	1.01 ± 0.01a	0.96 ± 0.02c	0.95 ± 0.02c	0.63 ± 0.62a	1.06 ± 0.16a	1.08 ± 0.03a	1.20 ± 0.04a	1.12 ± 0.16a	1.17 ± 0.14a
β-sitosterol	88.90 ± 0.01a	87.35 ± 0.05b	85.86 ± 0.08c	86.23 ± 0.22d	85.68 ± 0.01c	85.62 ± 0.16c	89.36 ± 0.09a	87.05 ± 0.23b	84.68 ± 0.43c	86.32 ± 0.20bd	85.92 ± 0.34d	86.83 ± 0.16d	85.58 ± 0.50a	83.11 ± 0.66b	82.86 ± 0.20b	82.82 ± 0.13b	84.65 ± 0.29c	86.06 ± 0.12a
sitostanol	0.79 ± 0.02a	0.70 ± 0.03b	0.60 ± 0.02c	0.48 ± 0.00d	0.48 ± 0.01d	0.47 ± 0.00d	0.67 ± 0.02a	0.58 ± 0.01b	0.50 ± 0.00c	0.46 ± 0.02c	0.44 ± 0.06c	0.42 ± 0.01c	0.51 ± 0.09a	0.45 ± 0.01ab	0.33 ± 0.07ab	0.36 ± 0.02ab	0.37 ± 0.09ab	0.31 ± 0.01b
Δ5-avenasterol	2.93 ± 0.04a	4.46 ± 0.10b	5.87 ± 0.06cd	5.65 ± 0.22c	6.07 ± 0.00d	6.42 ± 0.09e	2.74 ± 0.13a	4.37 ± 0.08b	5.53 ± 0.25c	4.29 ± 0.12b	4.94 ± 0.08d	4.25 ± 0.23bd	6.53 ± 0.09a	7.81 ± 0.21b	8.13 ± 0.08b	7.78 ± 0.23b	6.65 ± 0.03a	5.28 ± 0.08c
Δ5,24-stigmastadienol	0.62 ± 0.03a	0.76 ± 0.02c	0.67 ± 0.05ab	0.77 ± 0.01cd	0.84 ± 0.01d	0.70 ± 0.05bc	0.57 ± 0.03a	0.77 ± 0.02b	0.81 ± 0.07b	0.61 ± 0.05c	0.61 ± 0.05c	0.51 ± 0.01c	0.90 ± 0.07a	1.17 ± 0.18b	1.11 ± 0.04ab	1.07 ± 0.03ab	0.90 ± 0.01a	1.00 ± 0.00ab
Δ7-stigmastenol ^b^	0.43 ± 0.01a	0.41 ± 0.01a	0.44 ± 0.04a	0.42 ± 0.03a	0.41 ± 0.01a	0.33 ± 0.01b	0.49 ± 0.05a	0.51 ± 0.03a	0.54 ± 0.00a	0.36 ± 0.03b	0.36 ± 0.03b	0.34 ± 0.02b	0.39 ± 0.04ab	0.40 ± 0.04a	0.32 ± 0.03bce	0.30 ± 0.01ce	0.26 ± 0.03e	0.37 ± 0.01abc
Δ7-avenasterol	0.72 ± 0.04a	0.91 ± 0.01bc	0.99 ± 0.01cd	1.02 ± 0.08d	1.00 ± 0.02cd	0.88 ± 0.01b	0.83 ± 0.01a	1.05 ± 0.03b	1.07 ± 0.06b	0.77 ± 0.01ac	0.70 ± 0.01cd	0.64 ± 0.03d	0.84 ± 0.00ab	0.92 ± 0.02b	0.92 ± 0.03b	0.80 ± 0.06a	0.65 ± 0.06c	0.67 ± 0.00c
app. β-sitosterol ^b^	94.30 ± 0.04a	94.29 ± 0.05a	93.99 ± 0.03b	94.04 ± 0.04b	94.02 ± 0.01b	94.13 ± 0.02c	94.36 ± 0.10a	93.83 ± 0.11b	**92.53 ± 0.11c**	**92.69 ± 0.02cd**	**92.87 ± 0.13d**	93.00 ± 0.04d	94.15 ± 0.01a	93.60 ± 0.12bd	93.50 ± 0.03bc	93.23 ± 0.14c	93.69 ± 0.18bd	93.83 ± 0.11d
total sterols ^b^	1764 ± 3a	1662 ± 20b	1814 ± 1c	1694 ± 33b	1740 ± 16a	1764 ± 2a	1595 ± 6a	1411 ± 8a	1339 ± 10a	1482 ± 10a	1314 ± 190a	1529 ± 24a	1262 ± 63a	1282 ± 95a	1303 ± 75a	1205 ± 54a	1486 ± 12b	1706 ± 49b

Values are mean ± standard deviation (*n* =2). ^a^ Different letters for each parameter indicate significant statistical differences (*p* < 0.05) among picking dates for each crop year (a–f). ^b^ Limits established by the current regulation: total sterols ≥ 1000 mg/kg; cholesterol ≤ 0.5%; brassicasterol ≤ 0.1%; campesterol ≤ 4.0%; stigmasterol ≤ campesterol; Δ7-stigmastenol ≤ 0.5%; app. β-sitosterol ≥ 93.0%. Values in bold: non-compliance. Not detected = nd.

**Table 6:** Evolution of sterol composition (%) and total sterols (mg/kg) according to picking date ^a^ for clone “Std” in the crop years 2017, 2018, and 2019.

	Clone “Std”(C-Std)																	
	Crop year 2017						Crop year 2018						Crop year 2019					
Parameter	4 Oct	18 Oct	7 Nov	22 Nov	29 Nov	13 Dec	1 Oct	15 Oct	28 Oct	11 Nov	25 Nov	11 Dec	4 Oct	18 Oct	7 Nov	22 Nov	29 Nov	13 Dec
ripening index	1.6	3.4	4.0	4.1	4.5	5.4	1.1	2.0	2.8	3.3	3.3	4.0	2.0	3.0	3.6	4.4	4.7	5.9
cholesterol ^b^	0.09 ± 0.01a	0.09 ± 0.01a	0.08 ± 0.01a	0.06 ± 0.01a	0.06 ± 0.01a	0.07 ± 0.00a	0.09 ± 0.05a	0.09 ± 0.01a	0.07 ± 0.01a	0.15 ± 0.00a	0.10 ± 0.01a	0.08 ± 0.04a	0.06 ± 0.00a	0.06 ± 0.01a	0.08 ± 0.01a	0.08 ± 0.02a	0.10 ± 0.02a	0.05 ± 0.03a
brassicasterol ^b^	nd	nd	nd	nd	nd	nd	nd	nd	nd	nd	nd	nd	nd	nd	nd	nd	nd	nd
24-methylene-cholesterol	0.05 ± 0.01a	0.05 ± 0.00a	0.06 ± 0.01a	0.07 ± 0.01a	0.05 ± 0.00a	0.08 ± 0.01a	0.04 ± 0.00a	0.07 ± 0.01abc	0.06 ± 0.01ab	0.08 ± 0.02bc	0.12 ± 0.00d	0.10 ± 0.01cd	0.10 ± 0.04a	0.09 ± 0.02a	0.09 ± 0.02a	0.12 ± 0.01a	0.13 ± 0.00a	0.11 ± 0.04a
campesterol ^b^	3.21 ± 0.01a	2.99 ± 0.01b	3.06 ± 0.00c	3.04 ± 0.00d	2.99 ± 0.00b	2.94 ± 0.01e	3.46 ± 0.00a	3.17 ± 0.01b	3.16 ± 0.02bc	3.12 ± 0.01c	3.16 ± 0.02bc	3.33 ± 0.00d	3.44 ± 0.02a	3.04 ± 0.02b	2.77 ± 0.05c	2.88 ± 0.00d	2.92 ± 0.02d	2.88 ± 0.04d
campestanol	0.46 ± 0.00a	0.43 ± 0.01b	0.42 ± 0.00b	0.34 ± 0.01c	0.31 ± 0.01d	0.33 ± 0.01cd	0.39 ± 0.01a	0.42 ± 0.04a	0.41 ± 0.00a	0.41 ± 0.00a	0.42 ± 0.01a	0.40 ± 0.05a	0.49 ± 0.02a	0.37 ± 0.09a	0.43 ± 0.02a	0.33 ± 0.03a	0.36 ± 0.02a	0.33 ± 0.04a
stigmasterol ^b^	0.55 ± 0.00a	0.65 ± 0.00b	0.85 ± 0.00c	0.81 ± 0.02d	0.88 ± 0.02e	0.74 ± 0.01f	0.72 ± 0.02a	0.86 ± 0.11a	1.90 ± 0.05b	2.75 ± 0.03c	2.56 ± 0.00d	2.54 ± 0.03d	0.93 ± 0.06a	0.87 ± 0.06a	1.53 ± 0.06b	1.84 ± 0.03c	1.87 ± 0.01c	1.16 ± 0.07d
clerosterol	0.99 ± 0.00a	0.96 ± 0.00ab	0.95 ± 0.00ab	0.93 ± 0.05bc	0.92 ± 0.01bc	0.89 ± 0.00c	1.23 ± 0.03a	1.07 ± 0.13a	1.10 ± 0.05a	1.02 ± 0.08a	0.97 ± 0.01a	1.06 ± 0.02a	1.45 ± 0.29a	1.34 ± 0.06a	1.42 ± 0.65a	0.93 ± 0.00a	0.91 ± 0.04a	0.96 ± 0.09a
β-sitosterol	87.10 ± 0.15a	86.03 ± 0.02b	85.59 ± 0.02c	84.92 ± 0.06ª	85.99 ± 0.10b	85.79 ± 0.02c	88.03 ± 0.14a	85.63 ± 0.39b	84.89 ± 0.07c	84.27 ± 0.16d	84.87 ± 0.12c	85.63 ± 0.33b	84.12 ± 0.75ac	83.33 ± 0.22ab	81.87 ± 1.05b	82.68 ± 0.00ab	83.26 ± 0.17ab	85.50 ± 0.54c
sitostanol	0.63 ± 0.01a	0.47 ± 0.01a	0.43 ± 0.00a	0.38 ± 0.17a	0.40 ± 0.03a	0.37 ± 0.00a	0.73 ± 0.01a	0.58 ± 0.01b	0.49 ± 0.09bc	0.43 ± 0.03c	0.51 ± 0.01bc	0.42 ± 0.03c	0.59 ± 0.06a	0.45 ± 0.02b	0.40 ± 0.10bc	0.43 ± 0.02bc	0.34 ± 0.03bc	0.30 ± 0.04c
Δ5-avenasterol	4.76 ± 0.10a	5.95 ± 0.01b	6.10 ± 0.02bc	6.35 ± 0.25cd	5.80 ± 0.11b	6.47 ± 0.00d	3.17 ± 0.05a	5.68 ± 0.06b	5.68 ± 0.07b	5.58 ± 0.07bc	5.49 ± 0.03c	4.85 ± 0.05d	6.61 ± 0.19a	7.74 ± 0.00bd	8.37 ± 0.23c	7.84 ± 0.00d	7.42 ± 0.03b	6.01 ± 0.13e
Δ5,24-stigmastadienol	0.66 ± 0.02a	0.80 ± 0.01b	0.80 ± 0.00b	0.91 ± 0.04c	0.76 ± 0.00bd	0.74 ± 0.00d	0.83 ± 0.03a	0.98 ± 0.05a	0.84 ± 0.07a	0.80 ± 0.00a	0.69 ± 0.12a	0.56 ± 0.27a	1.05 ± 0.05a	1.21 ± 0.11a	1.29 ± 0.05a	1.23 ± 0.06a	1.11 ± 0.06a	0.99 ± 0.18a
Δ7-stigmastenol ^b^	0.49 ± 0.07a	0.47 ± 0.01a	0.51 ± 0.03a	**0.60 ± 0.01b**	**0.66 ± 0.02b**	0.51 ± 0.01a	0.52 ± 0.01a	0.45 ± 0.01b	0.40 ± 0.04bc	0.43 ± 0.02b	0.39 ± 0.03bc	0.35 ± 0.03c	0.37 ± 0.01a	0.41 ± 0.05ab	0.48 ± 0.03bc	0.52 ± 0.02c	0.52 ± 0.02c	0.61 ± 0.04d
Δ7-avenasterol	1.00 ± 0.00a	1.10 ± 0.01b	1.14 ± 0.01c	1.24 ± 0.03d	1.18 ± 0.01e	1.07 ± 0.02b	0.79 ± 0.00a	1.04 ± 0.04b	0.98 ± 0.05b	0.96 ± 0.01b	0.73 ± 0.02ac	0.65 ± 0.05c	0.79 ± 0.04a	1.09 ± 0.01b	1.27 ± 0.02c	1.12 ± 0.06b	1.02 ± 0.10b	1.03 ± 0.02b
app. β-sitosterol ^b^	94.14 ± 0.06a	94.21 ± 0.01a	93.87 ± 0.04ab	93.49 ± 0.47b	93.86 ± 0.00ab	94.26 ± 0.01a	93.99 ± 0.07a	93.93 ± 0.16a	92.99 ± 0.08b	**92.10 ± 0.03c**	**92.53± 0.01d**	**92.53 ± 0.07d**	93.83 ± 0.016a	94.07 ± 0.15a	93.35 ± 0.02b	93.11 ± 0.03b	93.04 ± 0.12b	93.76 ± 0.17a
total sterols ^b^	1294 ± 10a	1331 ± 4ab	1366 ± 4b	1748 ± 28c	1776 ± 4c	1772 ± 23c	1489 ± 31a	1487 ± 35a	1346 ± 8b	1314 ± 16bc	1279 ± 34c	1429 ± 18a	1281 ± 42a	1340 ± 35ab	1432 ± 25bc	1516 ± 94cd	1501 ± 40cd	1573 ± 11d

Values are mean ± standard deviation (*n* =2). ^a^ Different letters for each parameter indicate significant statistical differences (*p* < 0.05) among picking dates for each crop year (a–f). ^b^ Limits established by the current regulation: total sterols ≥ 1000 mg/kg; cholesterol ≤ 0.5%; brassicasterol ≤ 0.1%; campesterol ≤ 4.0%; stigmasterol ≤ campesterol; Δ7-stigmastenol ≤ 0.5%; app. β-sitosterol ≥ 93.0%. Values in bold: non-compliance. Not detected = nd.

**Table 7:** Evolution of sterol composition (%) and total sterols (mg/kg) according to picking date ^a^ for clones 4 and 7 in the crop years 2017, 2018, and 2019.

	Clone 4 (C-4)						Clone 7 (C-7)					
	Crop year 2018						Crop year 2018					
Parameter	1 Oct	15 Oct	28 Oct	11 Nov	25 Nov	11 Dec	1 Oct	15 Oct	28 Oct	11 Nov	25 Nov	11 Dec
ripening index	1.0	1.6	2.4	2.7	3.3	3.7	1.0	1.7	2.6	2.7	3.4	3.7
cholesterol ^b^	0.10 ± 0.01a	0.11 ± 0.01a	0.08 ± 0.03a	0.09 ± 0.01a	0.10 ± 0.00a	0.09 ± 0.01a	0.09 ± 0.02a	0.08 ± 0.00a	0.09 ± 0.01a	0.09 ± 0.01a	0.09 ± 0.00a	0.08 ± 0.01a
brassicasterol ^b^	nd	nd	nd	nd	nd	nd	nd	nd	nd	nd	nd	nd
24-methylene-cholesterol	0.03 ± 0.01a	0.04 ± 0.00ab	0.07 ± 0.01b	0.05 ± 0.01ab	0.10 ± 0.00c	0.06 ± 0.00b	0.02 ± 0.00a	0.04 ± 0.01ab	0.06 ± 0.00bc	0.06 ± 0.02bce	0.08 ± 0.00ce	0.10 ± 0.03e
campesterol ^b^	3.43 ± 0.01a	3.30 ± 0.05bc	3.17 ± 0.01c	3.37 ± 0.06ab	3.18 ± 0.02c	3.26 ± 0.02c	3.54 ± 0.03a	3.40 ± 0.03b	3.27 ± 0.01c	3.45 ± 0.01b	3.33 ± 0.01d	3.54 ± 0.02a
campestanol	0.41 ± 0.04a	0.42 ± 0.06a	0.50 ± 0.10a	0.46 ± 0.07a	0.46 ± 0.01a	0.44 ± 0.00a	0.40 ± 0.01a	0.42 ± 0.04a	0.43 ± 0.01a	0.38 ± 0.02a	0.41 ± 0.02a	0.40 ± 0.01a
stigmasterol ^b^	0.71 ± 0.01a	0.98 ± 0.02b	2.26 ± 0.02c	2.70 ± 0.04d	2.95 ± 0.02e	2.82 ± 0.02f	0.59 ± 0.04a	0.71 ± 0.00a	2.03 ± 0.01b	2.42 ± 0.26c	2.80 ± 0.04d	2.32 ± 0.05c
clerosterol	1.16 ± 0.07a	1.13 ± 0.02a	1.10 ± 0.15a	1.02 ± 0.02a	0.99 ± 0.02a	0.96 ± 0.01a	1.09 ± 0.04a	1.08 ± 0.02ab	1.03 ± 0.02bc	1.03 ± 0.00b	0.97 ± 0.00cd	0.95 ± 0.04d
β-sitosterol	88.75 ± 0.49a	86.72 ± 0.02b	84.70 ± 0.27c	86.00 ± 0.09d	84.92 ± 0.14c	85.83 ± 0.14d	88.98 ± 0.22a	87.72 ± 0.11b	85.78 ± 0.02c	86.85 ± 0.43d	85.84 ± 0.05c	87.34 ± 0.14bd
sitostanol	0.72 ± 0.01a	0.65 ± 0.03b	0.54 ± 0.06c	0.47 ± 0.02cd	0.36 ± 0.02e	0.42 ± 0.02de	0.75 ± 0.04a	0.59 ± 0.02b	0.48 ± 0.02c	0.46 ± 0.06cd	0.43 ± 0.04cd	0.39 ± 0.00d
Δ5-avenasterol	2.89 ± 0.07a	4.66 ± 0.07b	5.59 ± 0.08c	4.13 ± 0.12d	5.32 ± 0.06e	4.57 ± 0.12b	2.74 ± 0.00a	3.93 ± 0.01b	4.78 ± 0.00c	3.81 ± 0.09d	4.58 ± 0.05e	3.65 ± 0.03f
Δ5,24-stigmastadienol	0.54 ± 0.13a	0.57 ± 0.08a	0.65 ± 0.06a	0.59 ± 0.03a	0.65 ± 0.01a	0.58 ± 0.04a	0.59 ± 0.11a	0.76 ± 0.06a	0.71 ± 0.00a	0.51 ± 0.11a	0.56 ± 0.01a	0.52 ± 0.06a
Δ7-stigmastenol ^b^	0.51 ± 0.04a	0.50 ± 0.07a	0.47 ± 0.02ab	0.39 ± 0.0bc	0.35 ± 0.05c	0.33 ± 0.02c	0.48 ± 0.04a	0.44 ± 0.01ab	0.42 ± 0.01b	0.32 ± 0.02c	0.32 ± 0.00c	0.23 ± 0.02e
Δ7-avenasterol	0.67 ± 0.04a	0.86 ± 0.01b	0.84 ± 0.01b	0.73 ± 0.04c	0.61 ± 0.01a	0.63 ± 0.02a	0.66 ± 0.01a	0.77 ± 0.01b	0.85 ± 0.00c	0.61 ± 0.04d	0.57 ± 0.02d	0.47 ± 0.01e
app. β-sitosterol ^b^	94.08 ± 0.19a	93.75 ± 0.03b	**92.58 ± 0.20c**	**92.20 ± 0.07d**	**92.24 ± 0.01d**	**92.36 ± 0.05cd**	94.16 ± 0.11a	94.08 ± 0.04a	**92.78 ± 0.00b**	**92.66 ± 0.29bc**	**92.38 ± 0.03c**	**92.84 ± 0.14b**
total sterols ^b^	1363 ± 67a	1220 ± 7b	1158 ± 61b	1216 ± 56b	1191 ± 2b	1251 ± 13b	1456 ± 4a	1290 ± 31b	1255 ± 15b	1376 ± 8b	1365 ± 14b	1406 ± 63b

Values are mean ± standard deviation (*n* = 2). ^a^ Different letters for each parameter indicate significant statistical differences (*p* < 0.05) among picking dates for each crop year (a–f). ^b^ Limits established by the current regulation: total sterols ≥1000 mg/kg; cholesterol ≤ 0.5%; brassicasterol ≤ 0.1%; campesterol ≤ 4.0%; stigmasterol ≤ campesterol; Δ7-stigmastenol ≤ 0.5%; app. β-sitosterol ≥ 93.0%. Values in bold: non-compliance. Not detected = nd.

**Table 8:** Combined within-group correlations between discriminant variables and standardized canonical discriminant functions and eigenvalues.

Parameters	Function 1	Function 2
Δ5,24-stigmastadienol *^a^*	−0.469 *^b^*	−0.006
Δ5-avenasterol *^a^*	−0.398 *^b^*	0.064
24-methylene-cholesterol	−0.337 *^b^*	−0.213
*β*-sitosterol	0.256 *^b^*	0.108
sitostanol	0.140 *^b^*	0.013
Campesterol *^a^*	0.136 *^b^*	−0.077
total sterols *^a^*	−0.117	0.627 *^b^*
Stigmasterol *^a^*	0.192	−0.406 *^b^*
Δ7-avenasterol *^a^*	−0.125	0.374 *^b^*
app. *β*-sitosterol	−0.200	0.357 *^b^*
Δ7-stigmastenol	0.020	0.342 *^b^*
cholesterol	−0.079	−0.170 *^b^*
eigenvalues	6.014	3.395
% variance	63.9	36.1
canonical correlation	0.926	0.879

*^a^* Selected variable. *^b^* The highest absolute correlation between each variable and any discriminant function.

## Data Availability

Data are contained within the article.
